# Hypofractionated Radiotherapy in Localized, Low–Intermediate-Risk Prostate Cancer: Current and Future Prospectives

**DOI:** 10.3390/medicina59061144

**Published:** 2023-06-14

**Authors:** Maria Chiara Lo Greco, Giulia Marletta, Giorgia Marano, Alessandro Fazio, Emanuele Buffettino, Arianna Iudica, Rocco Luca Emanuele Liardo, Roberto Milazzotto, Pietro Valerio Foti, Stefano Palmucci, Antonio Basile, Francesco Marletta, Francesco Cuccia, Giuseppe Ferrera, Silvana Parisi, Antonio Pontoriero, Stefano Pergolizzi, Corrado Spatola

**Affiliations:** 1 Radiation Oncology Unit, Department of Biomedical, Dental and Morphological and Functional Imaging Sciences, University of Messina, 98122 Messina, Italy; marlettagiulia1@gmail.com (G.M.); giorgiamarano@gmail.com (G.M.); ema_bff@hotmail.it (E.B.); ariannaiudicaa@gmail.com (A.I.); silvana.parisi@unime.it (S.P.); apontoriero@unime.it (A.P.); stefano.pergolizzi@unime.it (S.P.); 2 Radiology I Unit, Department of Medical Surgical Sciences and Advanced Technologies “G.F. Ingrassia”, University of Catania, 95123 Catania, Italy; fazio.alessandro1993@gmail.com (A.F.); pietrofoti@hotmail.com (P.V.F.); spalmucci@unict.it (S.P.); basile.antonello73@gmail.com (A.B.); 3 Radiation Oncology Unit, Department of Medical Surgical Sciences and Advanced Technologies “G.F. Ingrassia”, University of Catania, 95123 Catania, Italy; lucaliardo@hotmail.com (R.L.E.L.); robertomilazz@hotmail.it (R.M.); 4 Radiotherapy Unit, A.O.E. Cannizzaro, 95126 Catania, Italy; francescomarletta1@gmail.com; 5 Radiotherapy Unit, ARNAS Civico Hospital, 90127 Palermo, Italy; francesco.cuccia@arnascivico.it (F.C.); giuseppe.ferrera@arnascivico.it (G.F.)

**Keywords:** prostate cancer, external beam radiotherapy, hypofractionation, ultrahypofractionation, stereotactic body radiotherapy, brachytherapy, proton beam radiotherapy, mp-MRI, MRI-LINAC, PSMA

## Abstract

At the time of diagnosis, the vast majority of prostate carcinoma patients have a clinically localized form of the disease, with most of them presenting with low- or intermediate-risk prostate cancer. In this setting, various curative-intent alternatives are available, including surgery, external beam radiotherapy and brachytherapy. Randomized clinical trials have demonstrated that moderate hypofractionated radiotherapy can be considered as a valid alternative strategy for localized prostate cancer. High-dose-rate brachytherapy can be administered according to different schedules. Proton beam radiotherapy represents a promising strategy, but further studies are needed to make it more affordable and accessible. At the moment, new technologies such as MRI-guided radiotherapy remain in early stages, but their potential abilities are very promising.

## 1. Introduction

Prostate cancer (PCa) is the second most common tumour and the fifth cause of death for cancer among men globally [1]. At the time of diagnosis, in developed countries such as the U.S., most patients have a clinically localized form of PCa, with the majority of them presenting with low- or intermediate-risk disease [2].

The EAU guidelines on PCa recommend managing patients with low-risk disease and a life expectancy >10 years with active surveillance, while patients with low-risk disease or asymptomatic intermediate-risk disease and a life expectancy <10 years should be treated with watchful waiting [3,4]. These approaches are fundamental to avoid over-treatment in patients with clinically insignificant PCa. Nevertheless, when indicated, radical treatment can still be performed, including different alternatives such as surgery, external beam radiotherapy (EBRT) and brachytherapy.

Nowadays, curative-intent EBRT is delivered using conventionally fractionated radiotherapy (CFRT) at a total dose of 76–80 Gy [5,6,7]. Albeit effective, this schedule requires a treatment course that lasts over 7–8 weeks, possibly increasing side effects and yielding a lower treatment efficacy [8].

In recent years, in the aim of shortening radiotherapy courses while maintaining excellent treatment outcomes, several randomized phase III trials have evaluated the efficacy of moderately hypofractionated and ultrahypofractionated radiotherapy in various scenarios of localized PCa treatment [9,10].

The scope of this paper is to provide a narrative review about the radiobiological findings that have contributed to the growing employment of hypofractionation in PCa care. Furthermore, the more recent literature and the best available evidence favouring the application of these treatment schemes in patients in the localized low–intermediate-risk category are accurately summarised. In addition, a panoramic of future prospectives, including MRI-guided radiotherapy, are presented.

## 2. Radiobiological Rationale for Hypofractionation

While most types of cancer benefit in terms of disease control and toxicity from conventionally fractionated schedules, other neoplasms have shown to be more sensitive to fractional doses [11]. In the context of CFRT, this heterogeneity in terms of biological effects can be faithfully explained by the linear quadratic (LQ) model, which describes the cell survival (CS) curves as a function of radiation dose [12].

It is traditionally accepted that radiation injury takes place inside the DNA through two types of damages: alpha (α) type, which is non-repairable and happens when adjacent DNA filaments are ruptured by a single ionizing accident, and beta (β) type, which is repairable with time and derives from the interaction between two proximal single-filament fractures caused by different ionizing accidents [13]. In the LQ model, α is the initial inclination of the CS bend and illustrates the cell intrinsic sensitivity to radiation. This coefficient is linearly dependent on dose. In addition, β is the curvature of the subsequent segment of the plot and is related to the dose-per-fraction and dose rate variability. This coefficient is proportional to the square of the dose, which leads to the model being quadratic. As a result, a higher α/β ratio is represented by a more linear CS curve, whereas a lower α/β ratio is related to a more inclined CS curve [14].

In practical terms, this ratio defines the fraction dose where both the alpha and beta components cause the same value of cell death. Therefore, early responding tissues or tumors with a rapid turnover are characterized by high α/β ratio and are less sensitive to fraction size or dose rate variations, while late-responding tissues or tumors with a slow turnover are characterized by low α/β ratio and are more susceptible to fraction size or dose rate increments [15].

Based on the above, it should be noted that the advantage of hypofractionation depends on the α/β values of the target in relation to the nearby tissues. Therefore, the choice of a proper α/β ratio is fundamental in correctly designing a hypofractionated radiation therapy (HFRT) schedule.

The α/β ratio of PCa has been discussed since the late 1990s, when Brenner and Hall first pointed out a value of 1.5 Gy with a 95% confidence interval (CI) ranging from 0.8 to 2.2 Gy. In the following years, Fowler et al. revised this analysis and obtained the same result, with a narrower CI ranging from 1.25 to 1.75 Gy [16,17]. Posterior studies have concluded that in addition to the fraction dose, the overall treatment duration should be accounted for. Specifically, moderately higher α/β values result when the time factor is considered in the calculation; therefore, many authors consider values ranging approximately from 2 to 2.7 Gy [11]. Furthermore, it has been suggested that further factors can add supplementary uncertainty to the choice of the α/β ratio during HFRT schedule planning, such as the presence of tumor oxygenation, hormonal therapies, cancer tissue heterogeneity and the risk group [18,19].

Regarding the biological effective dose (BED), in recent clinical studies, a value of 180–200 Gy has been used. Studies that used the highest BEDs reported more significant late effects.

Even if all the above calculations indicate an inherent ambiguity in the α/β ratio when applied to clinical practice, they all agree on a small value of the α/β ratio of PCain comparison to its surrounding organs (bladder and rectum values of 5.6 Gy and 3 Gy, respectively), supporting an appealing rationale to administer HFRT in PCa [10,19,20].

## 3. Moderately Hypofractionated Radiotherapy as a Definitive Treatment

### 3.1. Dose-Fractionation Schemes

In recent decades, radiation oncologists have shown great interest in seeking different fractionation schemes for localized PCa.

While CFRT schedules employ daily fractions of 1.8–2.0 Gy, the fractional doses used in hypofractionation is variable and arbitrary. It can be subdivided into the following categories:

- Moderate hypofractionation, with a fraction size of 2.4–3.4 Gy,
- Ultrahypofractionation, with a fraction size ≥ 5 Gy [21,22].

Fraction sizes of between 4 Gy and 5 Gy are instead barely used in PCa clinical practice.

The most relevant study on moderate hypofractionation for localized PCa is the CHHiP study, a randomized, non-inferiority, phase III trial, which recruited 3216 patients with localized PCa (pT1b–T3aN0M0). In this trial, all patients were casually allocated (1:1:1) to receive either CFRT (74 Gy delivered in 37 fractions) or one of two hypofractionated schemes (either 60 Gy delivered in 20 fractions or 57 Gy delivered in 19 fractions). In the hypofractionated schedules, the BED was quantified to be comparable to those in the conventional one, considering an α/β ratio of 2.4 Gy for the hypofractionated groups. The primary endpoint was the time to biochemical or clinical failure, with a critical hazard ratio (HR) of 1.208 at an average follow-up (FU) of 5.2 years. This study, which directly compared two different hypofractionated regimens to CFRT, demonstrated the non-inferiority of using a fraction size ranging from 60 Gy to 74 Gy, while the non-inferiority of using a fraction size ranging from 57 Gy to 74 Gy could not be claimed. Although acute bowel and bladder symptoms appeared earlier in the hypofractionated groups, compared to the control arm (4–5 weeks vs. 7–8 weeks), only 2% of patients experienced grade 3 or worse toxicities, and only 1% had a treatment time protraction of more than one week, supporting the feasibility of hypofractionated schedules. Five years after radiotherapy treatment, the incidence of grade 2 or greater bowel, bladder and sexual-clinician-reported toxicities were similar in all the groups of treatment. Based on the above, it can be deduced that the integration of hypofractionation with high-quality treatment methods offers an outstanding tumor control, lower toxicities and greater benefits for men with PCa compared to CFRT [23].

Other studies that evaluated the non-inferiority of moderate hypofractionated schedules include the PROFIT and RTOG 0415 trials.

The PROFIT trial is a multicenter, randomized, non-inferiority, phase III trial, which enrolled 1204 patients with intermediate-risk disease to perform either a treatment of 78 Gy in 39 fractions of 2 Gy or a treatment of 60 Gy in 20 fractions of 3 Gy. The primary outcome was biochemical failure. The non-inferiority margin was 7.5% (HR < 1.32). This study, at an average FU of 6 years, showed that the hypofractionated regimen had no less of a biochemical and clinical failure rate than the control group (85% in both arms), reporting, in addition, a lower late toxicity rate in the hypofractionated group [24].

Similarly, the RTOG 0415 trail is a randomized, non-inferiority, phase III trial, which recruited 1115 patients affected by low-risk disease. All patients were casually assigned (1:1) to receive either a treatment of 73.8 Gy in 41 fractions or a treatment of 70 Gy in 28 fractions. The primary scope of this trial was to analyze the differences between the two groups in terms of disease-free survival (DFS), including local and distant progression, biochemical recurrence or death from any cause. With regard to efficacy, it demonstrated the non-inferiority of using a treatment 70 Gy in 28 fractions compared to a treatment 73.8 Gy in 41 fractions, albeit that greater grades 2 and 3 gastrointestinal (GI) and genitourinary (GU) late toxicities were detected [25].

Finally, with the purpose of demonstrating the superiority of hypofractionation in comparison to conventional schemes, the Dutch HYPRO trial was performed. In this multicentric, randomized, open-label, phase III study, 820 men with intermediate–high-risk disease were assigned (1:1) to receive either a treatment of 78 Gy delivered in 39 fractions (5 days a week) or a treatment of 64.6 Gy delivered in 19 fractions (3 days a week). The primary endpoint was relapse-free survival (RFS). At an average FU of 5 years, no difference among the treatment arms was detected, highlighting the non-superiority of the hypofractionated scheme compared to CFRT [26,27].

The details of all the studies are summarized in Table 1.

Even if the patients enrolled in these trials belonged prevalently to low- and intermediate-risk groups, the high-risk group was adequately taken into account in the complexity of the studies; therefore, the evidence is considered to be high-quality for all categories. Based on the above, a solid consensus to perform moderate hypofractionation in patients eligible for EBRT has been achieved in all risk groups. Even if an optimal regimen cannot be determined, schedules of 60 Gy in 20 fractions and 70 Gy in 28 fractions are supported by the largest evidentiary base. Among these, the first is preferred [21].

### 3.2. The Use of the Spacer Technique in Hypofractionated Schedules

The anatomical relationship between the prostate gland and the anterior rectal wall makes the rectum susceptible to radiation damage, leading to an increased risk of exhibiting early and late GI toxicities.

Even if the majority of trials suggest that moderately hypofractionated radiotherapy is comparable to CFRT with respect to toxicities, pooled analyses have denoted higher GI adverse events after hypofractionated radiotherapy [28,29]. With the aim of avoiding damage to the rectum, the insertion of a rectal spacer balloon and the injection of a spacer into the perirectal fat have been recently investigated.

The use of biodegradable rectal spacer balloon in patients with intermediate-risk disease undergoing IMRT has been demonstrated to be a safe and effective method to spare the wall of the rectum from the effects of a higher dose to the prostate [30].

A slightly less invasive approach is the injection of biodegradable or non-biodegradable materials into the area between the prostate gland and the rectum, thus shifting away the rectal anterior wall from the high-radiation-dose zone. In this scope, different materials such as hydrogels, collagen and inflatable balloons have been investigated. Inflated balloons are not biodegradable and necessitate surgery to be removed, while collagen is not adequately supportive and decomposes too quickly. Therefore, nowadays, injectable hydrogel represents an appealing approach that is able to preserve the rectum during PCa hypofractionated radiotherapy due to its characteristic stability and biocompatibility [31].

Different studies have evaluated the clinical outcomes of patients who underwent spacer injection followed by hypofractionated radiotherapy.

A systematic review reported a lower radiation dosage to the rectum across several dosimetry levels. The study outlines a meaningful decrease in acute grade 1 diarrhea, late grade 1 and grade ≥ 2 rectal toxicities and urinary incontinence [32].

In addition, sexual function was also assessed before and after radiotherapy using the International Index of Erectile Function-5 (IIEF-5) scale, highlighting the possibility of maintaining sexual potency in patients, even after radiotherapy treatment. An Italian experience on 56 patients demonstrated the efficacy of hydrogel injections, concomitant to the use of intraprostatic fiducials, in maintaining erectile function after an HFRT schedule (60 Gy in 20 fractions, 5 days a week) in 62.5% of patients [33].

Furthermore, Applewhite et al. showed that hydrogel spacers can also be employed in a salvage setting after cryoablation [34].

The RPAH1 (hyaluronic acid for hypofractionated prostate radiotherapy) non-randomized, phase II study examined grade ≥ 2 GI late toxicity rates at 3 years after hypofractionated radiotherapy (62 Gy at 3.1 Gy per fraction) in patients who received a hyaluronic acid injection between the prostate and rectum. No grade 3 or 4 rectal toxicities were observed, the grade 2 rectal bleeding rate was less than 10% at 36-month FU and the 3-year late urinary toxicities were decreased [35].

Further results from phase III studies with a larger quantity of patients are awaited to assess the clinical benefit of spacer injection in hypofractionated extreme irradiation.

## 4. Ablative Stereotactic Body Radiotherapy: From a Seven- to a One-Fraction Regimen

Stereotactic body radiotherapy, also called ultrahypofractionation or extreme hypofractionation, is an innovative radiation approach capable of administering, with substantial precision, an elevated radiation dose (with a fraction size ≥ 5 Gy) to little and distinctly defined targets using either one (stereotactic radiosurgery) or a few fractions [36].

Even though a body of the literature suggests that 5 Gy represents the limit beyond which the LQ model is no longer applicable, the supporting data for this delivery method are quickly accumulating, with this method showing very promising results in terms of biochemical control and short- and long-treatment-related toxicity in low- and intermediate-risk-group patients [21,37,38].

With the aim of assessing RFS rates after SBRT, a pooled analysis from a multi-institutional consortium was performed. In this analysis, a total of 1100 patients with localized PCa were recruited in different prospective phase II clinical trials. At an average FU of 36 months, promising outcomes regarding both biochemical RFS and toxicity emerged [39].

Additionally, several randomized phase III trials have been recently performed to evaluate SBRT in comparison to CFRT and moderate hypofractionation in localized PCa.

The Scandinavian HYPO-RT-PC study is the first multicenter, open-label, randomized, controlled, non-inferiority, phase III study reporting the efficacy and side-effects of ultrahypofractionation compared to CFRT. In this trial, 1180 men with intermediate- and high-risk PCa were randomized (1:1) to receive either a treatment of 42.7 Gy in seven fractions or 78 Gy in thirty-nine fractions. The primary endpoint was PSA relapse, clinical failure, or both. After an average FU of 5 years, the non-inferiority of ultrahypofractionation compared to CFRT in terms of failure-free survival was demonstrated. Even though statistically significant differences in terms of acute toxicities and quality of life (QOL) were observed in the ultrahypofractionated arm, a posterior patient-reported QOL analysis demonstrated that 6 years after treatment conclusion, ultrahypofractionation was as well-tolerated as CFRT, supporting the application of ultrahypofractionation in intermediate- and high-risk groups [40,41].

In addition, the PACE-B trial, an international, open label, randomized, non-inferiority, phase III trial, recruited patients with low- or intermediate-risk disease from 37 centers in different countries (UK, Ireland, and Canada). In this study, eight hundred and seventy-four patients were casually allocated (1:1) to receive either a treatment of 78 Gy in thirty-nine fractions or a treatment of 62 Gy in twenty fractions as alternatives to a treatment of 36–25 Gy in five fractions in 1–2 weeks. The primary endpoint of the PACE-B trial was time to treatment failure (biochemical or clinical). At 24 months, SBRT was observed to be secure with low rates of adverse events. The results in terms of treatment failure are still pending [10,42].

To date, five- or seven-fraction SBRT regimens are considered valid treatment alternatives, with emerging data supporting schemes of fewer than five fractions. In this context, the most common fractionation schedule is a four-fraction regimen to a total dose of 38 Gy, which has demonstrated a modest toxicity rate and an excellent biochemical response [43,44].

The 2STAR phase II prospective trial investigated a treatment of 26 Gy in two fractions (one fraction per week) in thirty patients with low- and intermediate-risk disease. The primary endpoint was QOL using the Expanded Prostate Cancer Index Composite (EPIC) questionnaire. At a median FU of 49.3 months, no acute grade 3 GU and GI toxicity was observed. The rate of late grade 3 GU and GI toxicity was 3.3%, while the biochemical RFS rate was 94.8% [45].

The ONE-SHOT study, an ongoing phase I/II prospective, multicenter, single-arm trial, is evaluating the safety and efficacy of a single-fraction SBRT of 19 Gy in patients with low–intermediate-risk disease. In this trial, a diminution of the dose to the urethra down to 17 Gy was possible thanks to real-time electromagnetic motion control. The early outcomes of the phase I part (on six patients), which have been recently published, demonstrated the feasibility and tolerability of this schedule during the first 3 months with no grade 2 GI adverse events and with grade 2 GU adverse events in half of the patients [46].

The details of all the studies are summarized in Table 2.

Nowadays, due to insufficient evidence, SBRT should not be considered a standard of care and, specifically, delivering one to four fractions to the prostate should not be realized outside of clinical trials [47]. Furthermore, there are few studies suggesting the risk of developing a second primary cancer after SBRT (0.7% possibility of developing radiation-induced cancer at 5 years), but this risk is supposed to decrease thanks to the advent of highly conformal local treatments [48,49].

## 5. The Use of Hypofractionation in an Adjuvant/Salvage Setting

Despite recent improvements in PCa diagnosis and therapy, after any local treatment, from 30 to 60% of patients will present disease recurrence during their life [50,51].

Postoperative radiotherapy represents a valid approach in a multidisciplinary strategy aimed to decrease the risk of distant metastases and biochemical relapse and improve event-free survival. It can be performed either in an adjuvant or salvage setting [52,53,54,55].

Adjuvant radiotherapy is recommended for highly selected patients with an adverse pathology such as ISUP grade group 4–5 and pT3 with or without positive margins [3]. Salvage radiotherapy is recommended for patients who experience biochemical recurrence after a monitoring time, following radical prostatectomy (RP) [56].

In this setting, many questions remain unsolved, for example whether to offer a combination treatment or not, what is the most adequate clinical target volume to irradiate and what is the best radiation dose to deliver [3,57]. The ASTRO/AUA guidelines support a minimal dose of 64–65 Gy delivered with CFRT, based on the hypothesis that after RP the tumor burden is microscopic, and therefore, a lower dose is sufficient [58]. Nevertheless, while some retrospective analyses have indicated that dose escalation is able to improve biochemical control, other retrospective reports and smaller prospective trials using higher doses have not detected a relevant improvement. At present, a dose escalation of up to 70 Gy is being investigated by the SAKK 09/10 trial [59].

A major reason for concern when using hypofractionation after RP is radiation-induced toxicity. It must be noted that curative and postoperative radiotherapy implies important anatomical and dosimetric differences that could make hypofractionated approaches difficult to be applied in this context. After RP, a large portion of the bladder is shifted into the prostatic fossa, and the fascial plane alongside the anterior rectal wall is disrupted. As a result, postoperative radiation therapy plans contain larger bladder volumes compared to the definitive setting, making the ability to meet tolerance dose constraints more difficult with hypofractionation [60].

Based on the above, the impact of hypofractionation in a postoperative setting remains unclear.

To investigate moderate hypofractionation after RP, Cozzarini et al. conducted a single-institution analysis of 1176 patients treated with CFRT or hypofractionation (median dose: 2.5 Gy/fraction) in a postoperative setting, demonstrating a higher rate of short- and long-term GU toxicity for the hypofractionated schedule [61].

Massaccesi et al. performed a phase II trial on 49 men affected by PCa at high risk of relapse after RP or with biochemical relapse. In this study, 49 patients received hypofractionated IMRT with a simultaneous integrated boost (62.5 Gy at 2.5 Gy/fraction), while 52 patients were treated with CFRT [62]. The results showed comparable GI toxicity rates and an increased grade 2 GU toxicity rate in the CFRT arm.

Furthermore, the randomized, phase III NRG GU003 trial, recently published as an abstract, demonstrated that moderate hypofractionated radiotherapy was not inferior to CFRT with regards to late patient-reported GU or GI toxicity [63].

Currently, the SHARE trial, a prospective, randomized, multi-institutional study, is assessing the efficacy and toxicity of hypofractionated salvage radiotherapy in patients with biochemical recurrence after RP. In this study, patients are being casually assigned, with a 1:1 allocation, to treatments of 65 Gy at 2.5 Gy per fraction and 66 Gy at 2 Gy per fraction. The results of this trial are still pending [64].

According to the literature data regarding toxicity rates after prostate bed SBRT, acute ≥ G2 GI and GU toxicity rates vary from 0 to 50% and from 0 to 33.3%, respectively, while late ≥ G2 GI and GU toxicity rates vary from 0 to 11.5% and from 0 to 38.5%, respectively [50]. In terms of oncological results, to date, conclusive data comparing SBRT to CFRT or moderately fractionated radiotherapy to the prostate fossa are still missing.

Based on the lack of high-quality evidence, the conclusions that can be drawn are somewhat limited. Ultrahypofractionated radiotherapy to the prostate fossa is still considered experimental, and it should be performed only in clinical trials.

## 6. Valid Alternatives to Photon Beam EBRT: From Brachytherapy to Charged Particles

### 6.1. Brachytherapy

Brachytherapy is a radiation therapy technique that is able to deliver high doses of radiation to the target volume whilst simultaneously reducing the dose to the nearby tissues. It can be administered either in one session using low-dose-rate brachytherapy (LDR-BT) or over multiple treatment fractions using high-dose-rate brachytherapy (HDR-BT) [65].

Modern HDR-BT arose in the early 1990s as an innovative and encouraging approach for PCa care. Nowadays, it is performed using the radionuclide iridium-192 (192Ir), which is characterized by a half-life of 73.8 days and releases photons with a median energy of 380 keV, mainly through beta minus decay [66].

HDR brachytherapy as a monotherapy is considered to be more advantageous compared to LDR because clinicians are not exposed to radiation, the body of the patient is free from radioactivity material after therapy, the treatment duration is reduced from many months to several minutes, the dose can be determined prior to its delivery and the radiation dose distribution is improved [67].

In 2016, Yoshioka et al. presented mature data about HDR-BT as a monotherapy in patients in intermediate- and high-risk groups using 48 Gy in eight fractions, 54 Gy in nine fractions or 45.5 Gy in seven fractions over 4 to 5 days, demonstrating the safety and efficacy of these schedules [68].

In the same year, Jawad et al. investigated the toxicity profiles of three different HDR-BT schedules (38 Gy delivered in four fractions, 24 Gy delivered in two fractions and 27 Gy delivered in two fractions), concluding that they are all valid alternatives for the treatment of low–intermediate-risk PCa [69].

On the contrary, studies that assessed the use of a one-fraction approach (19–21 Gy) with the aim of sparing patients from multiple anesthesia events showed a notably higher rate of biochemical failure. The potential explanation for this phenomenon is that a one-fraction approach cannot be accurately represented by the LQ model. In this context, achieving tumor control could demand a higher dose than that estimated by traditional radiobiology models. [70].

In conclusion, the use of one-fraction HDR-BT as a monotherapy is limited to the context of clinical studies.

### 6.2. Proton Beam Hypofractionated Radiotherapy

Proton beam therapy (PBT) is a modern EBRT technique that is provided by charged particle beams and characterized by the capability of administering a homogeneous dose to the target, thus preserving the normal tissue in the neighborhood [71,72]. Differently from photons that release their energy near the entrance of the irradiated surface, protons liberate almost all the radiation dose at the end of the path, causing an event known as the Bragg peak. Before the Bragg peak, the deposited dose is about 30%; afterwards, the energy reduces nearly to zero. This sharp distal fall-off causes a reducing integral radiation dose to the organs at risk, thus helping to avoid side effects [73].

Considering the substantial investment necessary to perform PBT, nowadays, decreasing the cost and the number of treatment sessions represents an active area of research. During the last decade, numerous studies have evaluated the use of hypofractionated PBT for PCa. Different schedules have been explored, with fraction doses ranging from 2.5 to 7.6 Gy.

In 2017, Henderson et al. documented the 5-year outcomes from a prospective study about image-guided accelerated hypofractionated PBT performed on 215 patients with PCa. Patients with low-risk disease received a treatment of 70 Gy in 28 fractions, while patients with intermediate-risk disease received a treatment of 72.5 Gy in 29 fractions. The primary endpoints were the acute grade 3 or major treatment-related toxicity rate. At an average FU of 5.2 years, the biochemical and clinical disease progression rates were 95.9% in the overall group and 98.3% and 92.7% in the low- and intermediate-risk groups, respectively. The late grade 3 GU and GI or higher toxicity rates at 5 years were 1.7% and 0.5%, respectively [74].

In the following years, Grewal et al. conducted a phase II trial aimed at assessing the clinical and patient-reported outcomes for patients with low-intermediate-risk disease. In this trial, 184 enrolled patients received moderately hypofractionated PBT (70 Gy in 28 fractions). At an average FU of 49.2 months, the rates of overall biochemical-failure-free survival were 94.4%, 92.5% and 93.8% for the low-, favorable intermediate- and unfavorable intermediate-risk cohorts, respectively. The overall survival at 4 years was 95.8%, with no statistically significant differences in overall survival being observed across all subsets. The rates of acute grade 2 or higher GI and GU adverse events were 3.8% and 12.5%, respectively, while the 4-year incidence rates of long-term grade 2 GI and GU adverse events were 13.6% and 7.6%, respectively [75].

To investigate the feasibility of extreme hypofractionated PBT, Kubes et al. accrued two hundred patients with early-stage PCa to receive a treatment of 36.25 Gy in five fractions. At an average FU of 36 months, the late grade 2 GI and GU toxicity rates were 5.5%, and 4%, respectively. No grade 3 or higher adverse events were displayed. PSA relapse was observed in 1.08% of the patients belonging to the low-risk subset and in 6.5% of the patients belonging to the intermediate-risk subset [76].

In recent times, Vapiwala et al. performed a multi-center analysis on men with localized, low- and intermediate-risk PCa managed with moderately hypofractionated IMRT or PBT (2.5–3 Gy per fraction). The late grade 2 and grade 3 GU toxicity rates were 15.0% and 1.6%, respectively. The late grade 2 and grade 3 GI toxicity rates were 11.1% and 0.4%, respectively. The authors deduced that both strategies are secure and well tolerated [77].

Nowadays, even if further studies are required to establish the best fractionation schedule to use, hypofractionation represents a promising strategy to make PBT more affordable and available to more patients.

## 7. Future Prospectives: The Application of MRI in HFRT for Localized PCa

The employment of multiparametric MRI (mp-MRI) is quickly gaining popularity in the management of PCa due to its enhanced diagnostic potential and its capacity to merge functional and anatomical information. Although it is beyond the aim of this work, a concise overview about the diagnostic duties of mp-MRI in localized PCa will be given in this section to introduce its possible role as a game-changer for the radiation oncology community.

Furthermore, for sake of completeness, a brief mention about the role of hybrid imaging with prostate-specific membrane antigen (PSMA) PET-CT and PET-MRI will be given [3].

### 7.1. Mp-MRI and PSMA PET as Diagnostic Tools

Combining multiple MRI sequences can provide detailed information about the prostate gland such as the PI-RADS score, location and extent of suspicious lesions. The PI-RADS score, specifically, has shown to be a fundamental element in identifying patients eligible for early treatment and in providing risk stratification, thus foreseeing the risk of biochemical failure and distant metastases [78,79]. Furthermore, mp-MRI can also be used to guide biopsies, reducing the need for random sampling and improving accuracy [80,81].

Four sequences are recommended for mp-MRI, including T1- and T2-weighted images, diffusion-weighted imaging (DWI) and dynamic contrast-enhanced imaging (DCEI). T1-weighted imaging is primarily employed to study regional lymph nodes and bone structures and to detect biopsy-related hemorrhage. T2-weighted imaging provides well defined anatomical images of the prostate gland zonal architecture with optimal soft-tissue contrast. It can detect PCa, which appears hypointense upon imaging. Since DWI measures the casual movement of molecules of water within tissue, it is employed to detect cancerous lesions, which show a stronger inhibition of water molecule motion compared to healthy tissue. The ADC map, which reflects water mobility, is acquired by performing DWI with numerous magnetic gradient strengths, and it is helpful for discerning PCa. Combining DWI with T2-weighted imaging improves the sensitivity and specificity for identifying PCa and characterizing transition-zone tumors. DCEI is helpful when T2- weighted and DWI are indeterminate or impaired by artifacts and has a crucial function in assessing local recurrence after interventions that change the prostate morphology [80].

With the aim of improving the diagnostic accuracy of MRI, the possibility of combining mp-MRI with ultrasound-fusion-guided biopsies has been investigated, resulting in a higher possibility of detecting clinically relevant PCa than using mp-MRI alone [79].

Furthermore, the ability of PSMA PET-CT and PSMA PET-MRI to distinguish high-grade from low-grade PCa has been investigated. In this regard, it has been shown in an interim analysis that the addition of a PSMA PET-CT-targeted biopsy to mp-MRI could significantly improve the detection of PCa and help the treatment-planning process [82,83,84]. Nowadays, it is strongly believed that 68Ga- or 18F-labeled PSMA PET-CT and PSMA PET-MRI represent a valid diagnostic instrument that is able to improve the management of many patients with both primary and recurrent PCa [85].

In a therapeutic setting, PSMA has also recently emerged as an interesting therapeutic target, supporting the basis for theranostics and promoting the notion of precision medicine [86].

### 7.2. Potential Role of MRI in Radiotherapy Treatment Planning and Delivering

The advantages of integrating MRI into the radiotherapy treatment course have been described since early 1980s. The benefits include an improved soft tissue contrast, a lack of ionizing radiation and the possibility of obtaining non-invasive functional and real-time imaging during the radiotherapy treatment. So far, the majority of the experiments of using MRI in radiotherapy have been about target delineation [87].

According to the emerging evidence that suggests that delivering an increased dose to the dominant intraprostatic lesion (DIL) can enhance disease control, the ability of mp-MRI to define the DIL has become a crucial point for radiation oncologists.

Based on the above, the Hypo-FLAME multicenter phase II trial was performed with the aim of investigating the safety of SBRT together with a simultaneous integrated microboost to the macroscopic lesion. This trial accrued one hundred patients with intermediate–high-risk PCa who received a treatment of 35 Gy in five fractions (one fraction per week) to the entire prostate gland with an integrated boost up to 50 Gy to lesion defined by mp-MRI. The primary endpoint of the study was acute toxicity related to treatment. Even if the potential benefits in terms of tumor control still need to be assessed, this trial showed a manageable cumulative incidence of acute GU and GI toxicity [88,89].

In addition to the implementation of target delineation, the latest technological advancements have led to the development of MRI–radiotherapy hybrid systems (Linacs equipped with on-board MRI imaging), enabling the performance of MRI-guided radiotherapy [90]. The introduction of these hybrid machines, along with the possibility of allowing an adjustment to the daily treatment plan, represents a potential revolutionizing tool. Although the use of this technology can entail a longer single-session duration, thus increasing the risk of intra-fraction motion, it can significantly reduce the inter-fraction variability, which represents a crucial issue in extreme hypofractionated schedules. Nowadays, even if MRI-guided radiotherapy is still in its early stages, its potential benefits in terms of dose escalation and reducing toxicity are very promising.

Currently, it remains resource- and time-intensive to administer and is not yet widely accessible. Before claiming any clear benefits of MRI-guided radiotherapy, further prospective randomized clinical trials and extensive clinical validations are required, although the theoretical potential of this technology is vast [91].

## 8. Conclusions

In developed countries, at the time of diagnosis, most PCa patients have clinically localized low–intermediate-risk disease. With the aim of avoiding over-treatment, the EAU guidelines on PCa recommend managing patients with clinically insignificant disease with watch-and-wait or active surveillance. Nevertheless, many patients are still eligible for radical, intense approaches, including surgery or radiotherapy.

Regarding radiotherapy, a solid consensus has been achieved in all risk groups to perform moderate hypofractionation, with a treatment of 60 Gy in 20 fractions being the most preferred schedule. With regard to ultrahypofractionation, at the moment, insufficient evidence is at our disposal, so it should not be considered as a standard of care.

Alternatively, HDR-BT can be administered according to different schedules with multiple fractions. In addition, PBT can be considered as an alternative even if further studies are needed to make it more affordable and accessible. At the moment, technologies such as MRI-guided radiotherapy remain in their early stages, but their potential abilities are very promising.

## Figures and Tables

**Table 1:** Prospective trials comparing conventionally fractionated radiotherapy to moderately fractionated radiotherapy.

Authors	Name	Type	Patients	Arms	Primary Endpoint	Follow Up	Results
Dearnaley, D. et al. [23]	CHHiP	Randomized, non-inferiority	3216	2 Gy × 37 Fx 3 Gy × 20 Fx 3 Gy × 19 Fx	Time to biochemical or clinical failure	62.4 months	88.3% 90.6% 85.9%
Catton, C.N. et al. [24]	PROFIT	Randomized, non-inferiority	1204	2 Gy × 39 Fx 3 Gy × 20 Fx	Time to biochemical or clinical failure	72 months	85% 85%
Lee, W.R. et al. [25]	RTOG 0415	Randomized, non-inferiority	1115	1.8 Gy × 41 Fx 2.5 Gy × 28 Fx	DFS	69.6 months	85.3% 86.3%
Incrocci, L. et al. [27]	HYPRO	Randomized, superiority	820	2 Gy × 39 Fx 3.4 Gy × 19 Fx	RFS	60 months	77.1% 80.5%

**Table 2:** Prospective trials evaluating ultrahypofractionation.

Authors	Name	Type	Patients	Arms	Primary Endpoint	FU	Results
Widmark, A. et al. [40]	HYPO-RT-PC	Randomized, non-inferiority	1180	2 Gy × 39 Fx 6.1 Gy × 7 Fx	Time to PSA relapse or clinical failure	60 months	84% 84%
Brand, D. h. et al. [42]	PACE-B	Randomized, non-inferiority	874	2 Gy × 39 Fx 3.1 Gy × 20 Fx 5–7.2 × 5 Fx	Time to biochemical or clinical failure	Still awaiting	Still awaiting
Alayed, Y. et al. [45]	2STARS	Single group	30	12 Gy × 2Fx	QOL and minimal clinically important change	49.3 months	EPIC: Urinary 1.1 Bowel 1.0 Sexual 3.8
Zilli, T. et al. [46]	ONE SHOT	Single group	6	19 Gy × 1 Fx	Clinical performance and progression free survival	Still awaiting	Still awaiting

## Data Availability

Not applicable.
